# Numerical Modeling of Orthogonal Cutting in 3D-Printed PEEK: Process Parameters and Simulation Validation

**DOI:** 10.3390/polym18212639

**Published:** 2026-10-24

**Authors:** David Gómez-García, Jose Díaz-Álvarez, George Youssef, Antonio Díaz-Álvarez

**Affiliations:** 1 Department of Mechanical Engineering, University Carlos III of Madrid, Avd. de la Universidad 30, Leganés, 28911 Madrid, Spain; 2 Experimental Mechanics Laboratory, Mechanical Engineering Department, San Diego State University, 5500 Campanile Dr, San Diego, CA 92182, USA

**Keywords:** PEEK, micromachining, numerical modeling, orthogonal cutting, damage, temperature

## Abstract

Additive manufacturing (AM) is rapidly transforming industrial production by offering a low-waste and highly customizable alternative to traditional subtractive methods. However, tight dimensional tolerances and complex geometries often require post-process machining of 3D-printed components. Among high-performance thermoplastics, polyether-ether-ketone (PEEK) is preferred for its thermal stability and mechanical strength. A key novelty of this study addresses a critical gap in the literature: while some analyses of PEEK machining consider cutting and pressure parameters, few documented instances examine unreinforced PEEK at high cutting speeds. This knowledge gap is highly relevant because the absence of reinforcement and high cutting speeds interact through temperature-induced thermal softening. Utilizing a validated finite element model in ABAQUS, the research presents a comprehensive numerical investigation of the orthogonal micromachining of 3D-printed PEEK, analyzing the influence of printing parameters (building orientation and strategy) alongside machining variables (cutting speed and depth of cut). The study identifies critical anisotropic effects, where specific build orientations, particularly −45°, maximize mechanical resistance and heat generation. Conversely, multi-layer stacking sequences (e.g., 0°/90° and 45°/−45°) are shown to enhance structural rigidity and improve surface finish by optimizing chip formation mechanics and reducing interfacial temperatures. These findings provide a consistent framework for optimizing the post-processing of additively manufactured advanced polymers.

## 1. Introduction

Traditionally, many manufactured parts are fabricated using material-removal processes (also known as subtractive manufacturing), which generate significant waste and require additional costly steps to reduce their environmental impact, such as recycling. Optimization of manufacturing processes has changed these procedures to reduce their ecological footprint and costs, facilitating the emergence of new manufacturing technologies [1,2]. Additive manufacturing (AM), commonly referred to as 3D printing, is an emerging manufacturing process in which raw material is deposited, usually in layers, from the ground up to form a final three-dimensional (3D) component or part. 3D printing has gained relevance over the years because of its low cost, low waste generation, versatility, and ability to achieve complex geometries, among others [3]. Because of these advantages, 3D printing is widely used in construction, aerospace, and biomedical applications (e.g., prosthetics) [4,5]. In short, 3D printing processes are infiltrating several industrial domains and accelerating the bench-to-market development cycle.

3D printing encompasses several additive manufacturing approaches, depending on the feedstock form (powder, pellets, filaments, etc.) and physical state (liquid or solid). Fused filament fabrication (FFF) is among the most widely used 3D printing approaches, specifically after the expiration of the original patent. In this process, spooled polymer filaments are extruded through a heated nozzle above their melting temperature and deposited layer by layer on a heated bed to achieve the required geometry [6]. The printing parameters used during the manufacturing process substantially influence the final performance of the parts, including nozzle and bed temperature [7,8], printing speed, and printing direction [9,10].

A key class of materials ubiquitous in 3D printing is thermoplastic polymers, due to their relatively low melting temperatures and tolerance to heating and cooling cycles [11]. The most widespread thermoplastics in FFF-AM include acrylonitrile butadiene styrene (ABS), polylactic acid (PLA), high-density polyethylene (HDPE), polyamide (PA), polyetherimide (PEI), poly-ether-ketone-ketone (PEKK), or poly-ether-ether-ketone (PEEK) [12,13,14]. Based on their applications and characteristics, these polymers fall into three categories: standard (PLA and ABS), engineering (HDPE and PA), and advanced polymers (PEI, PEKK, and PEEK). PEEK stands out among its competitors as one of the best options because of its high-temperature capabilities, enhanced mechanical properties, and biocompatibility, making it a strong choice for the medical field for personalized bone and dental prostheses [15,16,17,18,19,20,21].

Despite PEEK’s additive processability and mechanical performance, some applications may still require post-processing machining to finalize the part for deployment. For example, thermal annealing (isothermal soak above the glass transition temperature) is imperative for AM-processed PEEK parts and components to erase thermal processing history and reorganize the molecular polymer composition to form crystals, thereby strengthening mechanical behavior [22,23,24]. In other words, thermal annealing is usually used to enhance material properties through crystallization, depending on thermal soak conditions and duration [25,26]. Furthermore, augmenting AM with traditional subtractive manufacturing approaches, e.g., material removal, is also imperative to meet requirements for tight tolerances or improved surface quality [18,27,28]. In material-removal post-processing steps, cutting speed and feed rate strongly influence machining forces [29], tool wear [30], and final mechanical behavior. Notably, printing orientation also influences finishing steps, such as surface finish, by altering material-breaking behaviors such as delamination [31] and chip formation [32]. Hence, a synergistic focus of this research study is to underscore the interrelation between the machining parameters of 3D-printed PEEK.

The high associated costs and the time required to develop sufficient knowledge to comprehend the behavior of emerging materials have raised the need for alternative techniques, such as numerical modeling. Holistic analysis of machining processes, such as milling [33], turning [34], or drilling [35], is also computationally prohibitive because it requires explicit dynamics, which is among the most time-consuming analyses within the finite element method (FEM) framework. Hence, optimizing and strategically simplifying the computational framework is imperative to ensure this analysis keeps pace with the complexities of finishing steps in 3D-printed parts and components. For example, the tool–workpiece interaction can be divided into two types of cutting, depending on the angle between the cutting direction and the filament printing orientation: oblique cutting and orthogonal cutting. In other words, this interaction can be expressed as a combination of different cutting processes based on tool heading as a function of filament orientation [36,37,38]. Therefore, controlling tool–workpiece interaction can significantly reduce computational costs, as the numerical model of the complex drilling process can be readily divided into sequential orthogonal cutting steps at reduced computational penalty. The existing literature offers limited research on the microscale orthogonal cutting machining process of PEEK, despite evidence of focus on orthogonal cutting simulation of carbon fiber-reinforced PEEK [39] and other materials [40]. Whilst there are analyses of PEEK machining that take cutting and printing parameters into account, there are no recorded cases in which unreinforced PEEK is subjected to high cutting speeds; as discussed herein, this is of great importance given the relationship between the absence of reinforcement and high cutting speeds, due to the thermal softening induced by temperature effects. Barge et al. studied the influence of numerical parameters on modeling material removal during orthogonal cutting [41], highlighting numerical challenges in implementation, e.g., the influence of mesh density, which directly affects chip morphology. In general, the efficacy of computational models hinges on selecting mechanical properties as a function of the manufacturing process and on mesh optimization, which involves associated computational costs [42,43].

Therefore, the overarching objectives of this research study are to comprehensively investigate the machinability of 3D-printed PEEK using experimental and computational approaches. Thus, this study goes beyond the current focus on the mechanical and thermal behavior of 3D-printed PEEK [44,45] or the machinability of molded PEEK [46,47]. The approach in the current study hinges on testing and analyzing the influence of cutting speed (30, 60, and 90 m/min), depth of cut (50, 100, and 150 µm) and filament orientation (0°, 90°, 0°/90°, 45°, −45°, and 45°/−45°) on the machining process and final state of a manufactured workpiece, resulting in a total of 54 tests: one for each cutting speed, cutting depth, and printing direction. Furthermore, this research aims to analyze the machinability of 3D-printed PEEK using the finite element method and to validate the results previously obtained through the experimental tests performed by Gómez-García et al. [48].

## 2. Experimental Methodology

As previously reported by Gómez-García et al. [48], the samples were manufactured using a FUMAT PRO 410 3D printer from INTAMSYS (Shanghái, China), with a 1.75 mm diameter PEEK filament supplied by ENSINGER (Nufringen, Germany). Table 1 lists the parameters used for printing the test specimens. The printing parameters were selected in accordance with the manufacturer’s recommendations. Table 1 lists the parameters used for printing the test specimens.

**Figure 1:**
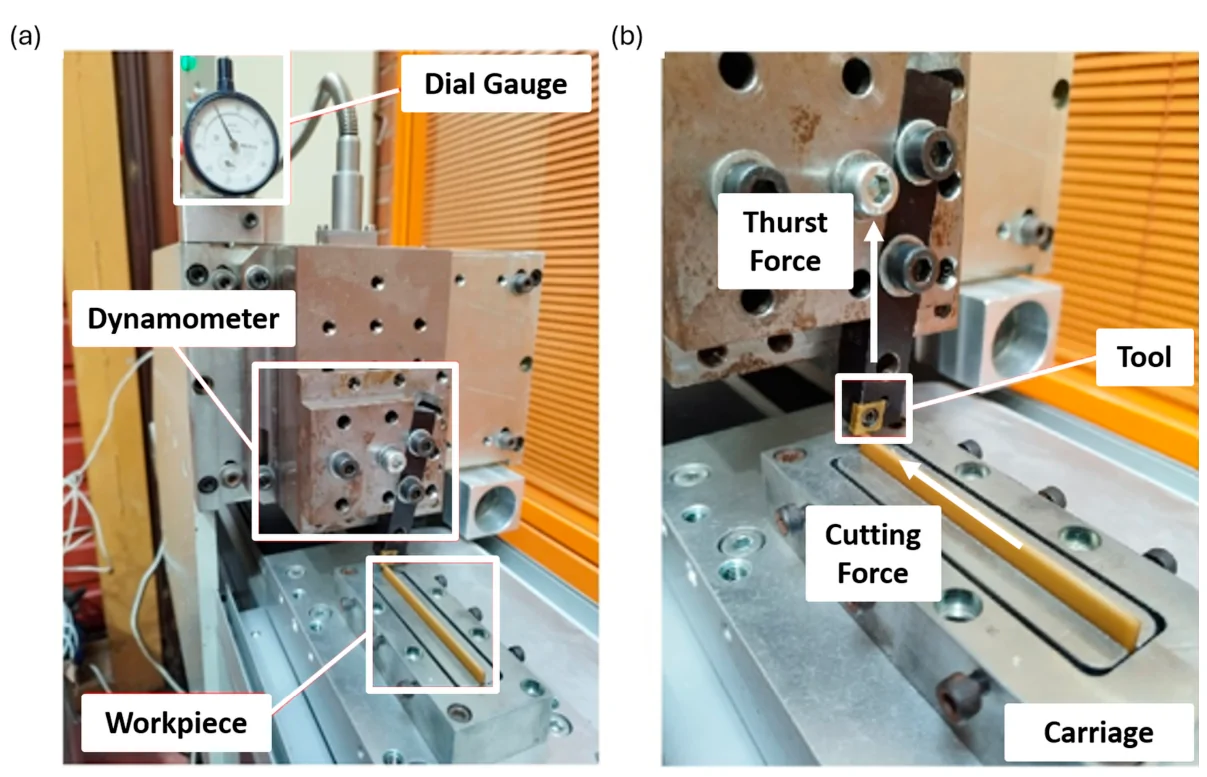
Orthogonal cutting machine explanatory scheme: (**a**) Overall view and (**b**) detailed photography of the workpiece–tool geometry.

All machining tests were performed using a Kistler 9257 B dynamometer (Winterthur, Switzerland) on a custom orthogonal cutting machine that accurately controlled cutting speed, carriage speed, and depth of cut using a dial gauge, as shown in Figure 1 [48].

Due to experimental constraints, a single test was performed per condition; while this allowed evaluation of overall trends, the lack of repeated trials is acknowledged as a limitation for statistical repeatability and variability.

**Figure 2:**
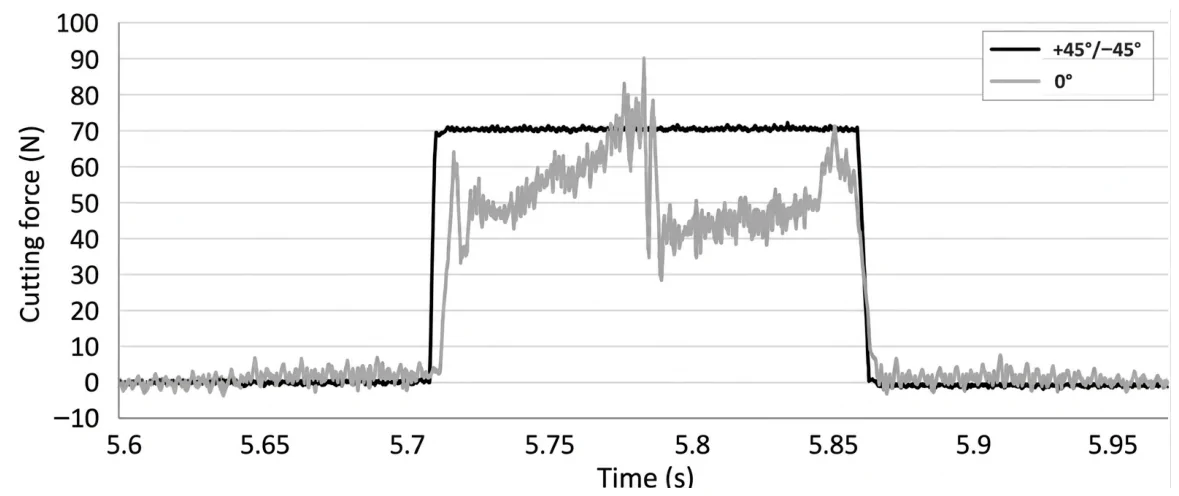
Force–−time signals for orthogonal machining at 30 m/min and 100 µm for 0° and 45°/−45° build orientations, adapted from Gómez-García et al. [48], Elsevier B.V., 2023.

A total of 18 tests were conducted as a function of each printing orientation (0° and 45°), cutting speed (30 m/min, 60 m/min, and 90 m/min), and depth of cut (50 µm, 100 µm, and 150 µm). The offset angles are defined based on the orientation of the tool–workpiece relative movement with respect to the filaments. This offset-angle nomenclature means that 0° corresponds to filaments parallel to the cutting force, and 90° to filaments parallel to the thrust force. Figure 2 recalls exemplary orthogonal machine results from [48], which are imperative for the upcoming discussion.

Previous experimental results demonstrated a strong relationship between printing orientation and depth of cut, as reflected in the forces recorded during machining [48]. The force-time histories resembled step functions for 45°/−45° printed samples (Figure 2). Alternatively, the force–time signals in the 0° cases captured the perturbations due to the tool–workpiece interactions, as shown in Figure 2. Furthermore, the printing filament orientation significantly affected the cutting forces, with the 45°/−45° orientation yielding the highest mean values. This difference also appeared in the material’s breaking behavior, as discussed in the forthcoming numerical modeling section.

The graph features vertical bars representing force values in Newtons (light gray for cutting force and dark gray for thrust force), categorized along the bottom axis by orientation (0° and ±45°), speed (30, 60, and 90 mm/min), and depth of cut (50, 100, and 150 μm).

**Figure 3:**
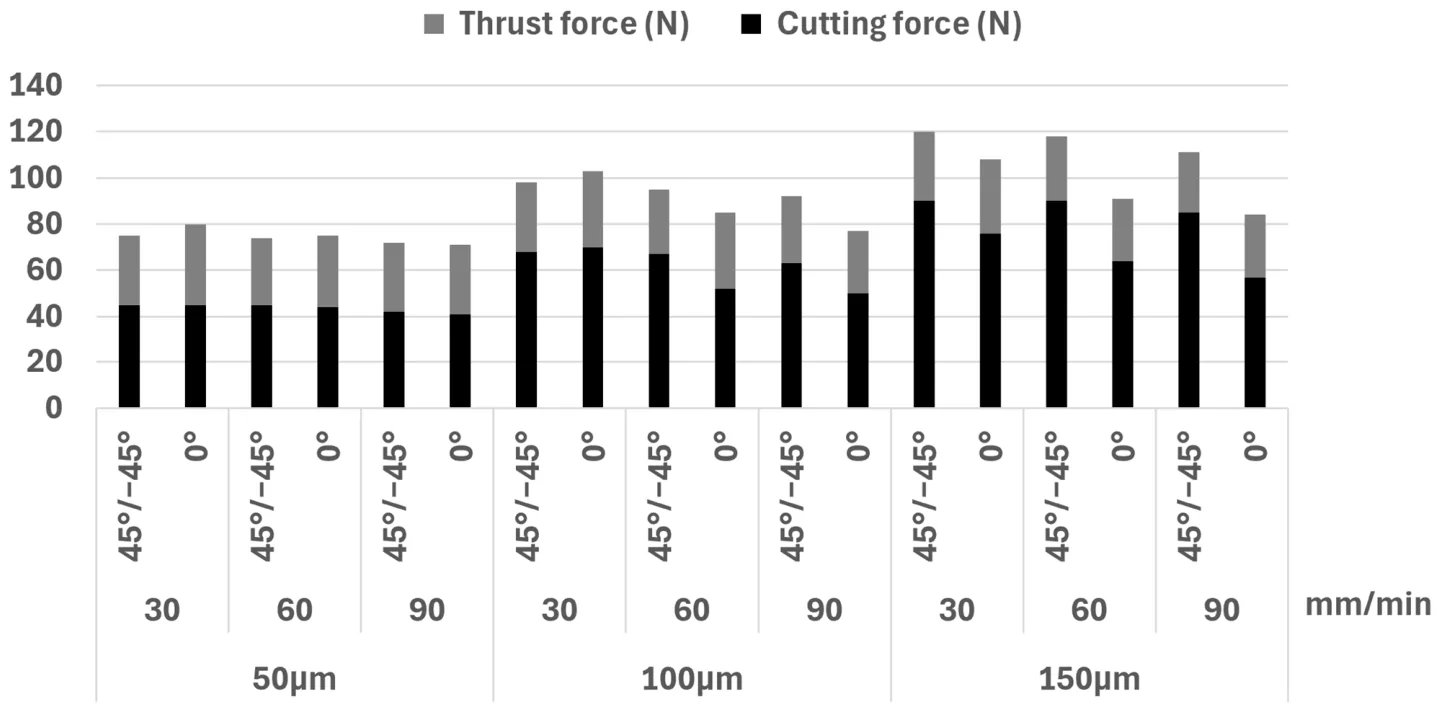
Cutting and thrust forces (on the ordinate axis in Newtons) from conducted orthogonal machining tests on PEEK samples as a function of cutting speed, depth of cut, and printing orientation (on the abscissa), adapted from Gómez-García et al. [48], Elsevier B.V., 2023.

Figure 3 summarizes the maximum cutting and thrust force signals as a function of orthogonal cutting parameters and sample orientation for 3D-printed PEEK. Cutting force increases with increasing depth of cut because it is proportional to the amount of material removed, leading to greater chip formation. Cutting speed has a slight impact on machining forces, with cutting force decreasing as cutting speed increases, especially at high cutting depths. This inverse relation is attributed to the strain-rate sensitivity of thermoplastic polymers such as PEEK and thermal softening as temperature increases at high cutting speeds [49]. A slight tendency is also observed in the thrust force, which decreases as cutting speed and depth of cut increase, with a marginal absolute variation of <7% throughout the tests. Therefore, the forthcoming computational framework focuses on cutting forces.

## 3. Numerical Modeling

The computational analyses were performed using ABAQUS 6.12 as the finite element solver, based on the definitions and settings specified in the model detailed in this section.

### 3.1. Definition

The experimental results summarized in the previous section on the orthogonal machining process are used here to validate the computational results. The focus is the interaction between the tool and the workpiece, which induces relative motion between them.

Coated carbide inserts supplied by SECO (grade TS2000, TiAl/TiAlN coating) with a designation CCMT09T304-F2 were used. The tool geometry is characterized by an 80° tip angle. The active cutting-edge geometry features a relief angle of 7°, a positive rake angle of 15°, and an edge preparation consisting of a 0.17 mm chamfer honing rounded with a 25 µm edge radius.

**Figure 4:**
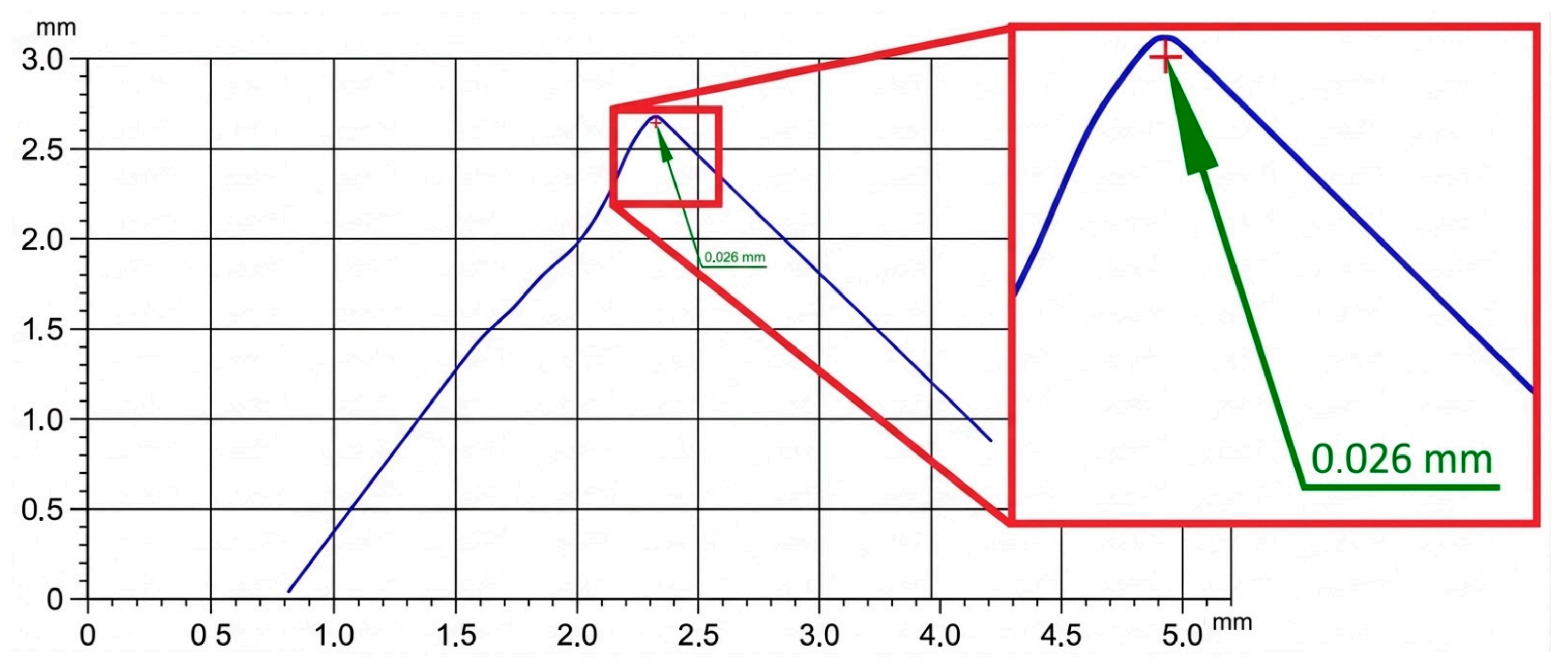
Region of interest of the tool geometry subtracted using the profilometer.

To accurately reproduce the experimental conditions, we measured the tool geometry used in the model with a Mahr Surf XC2 profilometer (Gotinga, Germany); the resolved tool shape (Figure 4), with a 0.026 mm cutting-edge radius, was imported into the ABAQUS environment as a point cloud.

**Figure 5:**
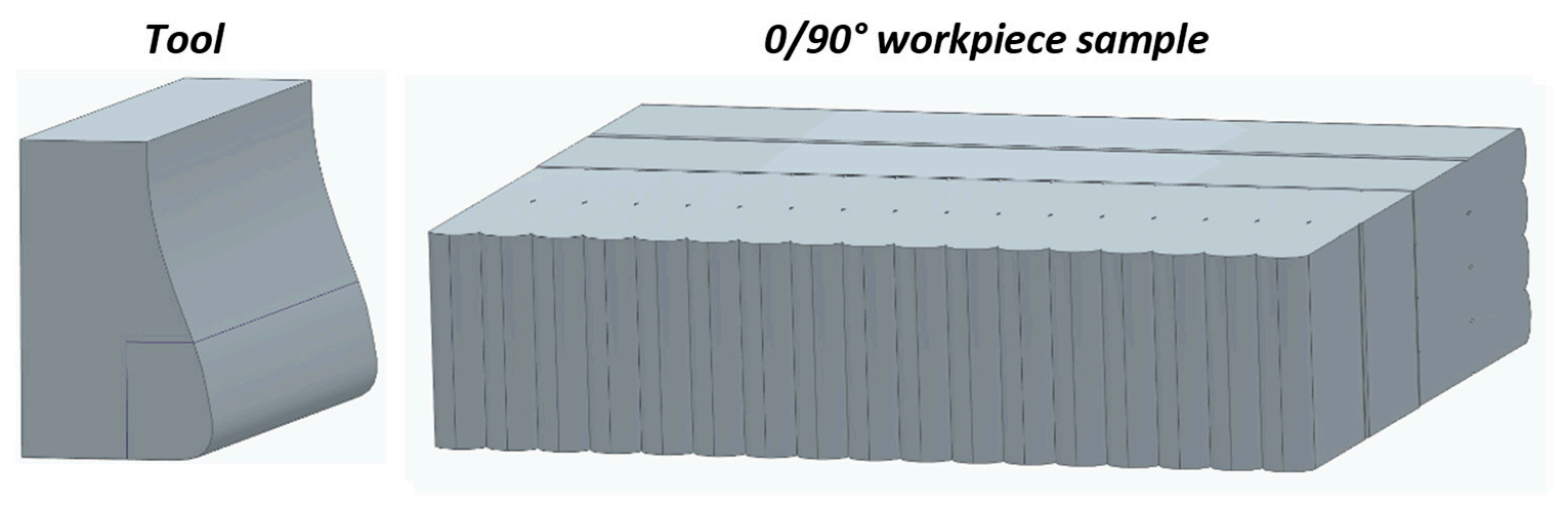
Tool and workpiece geometries modeled for the computational analysis using FEM.

The workpiece geometry is constructed from a CT scan of a 3D-printed PEEK sample, allowing precise replication of the solid topology, including the deposited filament and the shape of the interstitial ducts. The geometry was modeled by neglecting potential printing defects, such as micro-voids or interlayer lack of adhesion, as shown in Figure 5.

Printing defects were not modeled because including them would introduce uncertainty and variability that would alter the machining model results. Analyzing the impact of such defects is a separate research problem and beyond the scope of this work, which focuses on the cutting forces exerted during the drilling of 3D-printed PEEK.

For the numerical analysis, a refined mesh of around 2 million elements was established through a mesh sensitivity analysis, with a predominance of cubic elements. To model the relative motion between the workpiece and the tool, the rear and top surfaces of the tool were fixed, while a vertical displacement condition corresponding to the cutting speed was applied to the bottom surface of the workpiece.

### 3.2. Constitutive Models and Failure Criteria

#### 3.2.1. Tool

The tool used during the experiments was a TS2000 carbide tool with a TiAl/TiAlN coating provided by SECO. The material was defined in the elastic regime because its yield strength is higher than that of the machined PEEK samples. In other words, the induced stresses (*σ*) in the tool during orthogonal machining are always below the yield strength (*σ_y_*). Therefore, the linear elastic stress–strain relation (Equation (1)) governs the tool’s behavior in isotropic materials.

*σ* = *E* · *ε* (1)

To account for temperature, heat conduction (Equation (2)) was introduced through thermal conductivity, enabling heat transfer between elements within the same body.

*q* = *k* (*ϴ_A_* − *ϴ_B_*) (2)

where *q* and *k* are the heat transfer and conductivity, respectively, and *ϴ_A_* − *ϴ_B_* is the temperature gradient between two elements. Table 2 shows the main material parameters for the cutting tool based on previous research [50].

To model the thermal field generated during the machining process, the transient heat conduction governing equation based on Fourier’s Law was implemented:

$$\rho c_{p}\frac{\partial T}{\partial t}=\nabla \cdot \left(k\nabla T\right)+Q_{g}$$

where *ρ* is the material density, *c_p_* is the specific heat capacity, *k* represents the thermal conductivity tensor, *T* is the temperature, and *Q_g_* accounts for the volumetric heat generation rate arising from plastic deformation and frictional work at the tool–chip interface.

#### 3.2.2. Workpiece

The mechanical behavior of PEEK encompasses three regimes: elastic, plastic, and degradation. The elastic regime of PEEK also follows the linear elastic constitutive relationship in Equation (1), including the conductive heat transfer in Equation (2). When *σ > σ_y_*, the plastic regime starts; the constitutive behavior of PEEK is described by the Johnson–Cook model [51], which has been previously proven reliable for non-metallic materials [52,53]. The Johnson–Cook model (Equation (3)) captures the influence of strain hardening, strain rate, and temperature on the stress–strain behavior [51].

(3) $$\sigma \left({\bar{\varepsilon }}^{p},{\dot{\bar{\varepsilon }}}^{p},T\right)=\left[A+B\cdot {\left({\bar{\varepsilon }}^{p}\right)}^{n}\right]\cdot \left[1+C\cdot \ln\left(\frac{{\dot{\bar{\varepsilon }}}^{p}}{{\dot{\bar{\varepsilon }}}_{0}^{p}}\right)\right]\cdot \left[1-{\left(\frac{T-T_{0}}{T_{m}-T_{0}}\right)}^{m}\right]$$

where ${\bar{\varepsilon }}^{p},{\dot{\bar{\varepsilon }}}^{p}$ and *T* are the strain hardening, the strain rate, and the temperature, respectively. *A* is the yield strength at room temperature, *B* is the strain-hardening parameter, *C* is the strain-rate constant, *n* is the strain-hardening exponent, and *m* is the thermal-softening exponent. All the Johnson–Cook parameters are extracted from the experimental stress–strain curves. *T*_0_ and *T_m_* correspond to the room temperature and the melting temperature of the material [54]. The temperature rate during adiabatic and high-strain-rate loading, as presented by Zaera et al. [55], is as follows:

(4) $$\rho {\cdot C}_{p}\cdot ∆T=\beta \cdot {\dot{W}}^{p}\cdot ∆t\;$$

(5) $${\dot{W}}^{p}=\bar{\sigma }\cdot {\dot{\bar{\varepsilon }}}^{p}$$

where *ρ*, *C_p_*, *β*, and ∆*T* are the density, the specific heat at constant pressure, the Taylor–Quinney coefficient, and the temperature increment. ${\dot{W}}^{p}$, $\bar{\;\sigma }$ and ${\dot{\bar{\varepsilon }}}^{p}$ are the plastic work, the stress, and the equivalent plastic strain rate, respectively. Finally, the plastic regime ends when the damage parameter *ω_D_* in Equation (6) reaches unity, indicating damage initiation [56].

(6) $$\omega_{D}=\int \frac{d{\bar{\varepsilon }}^{p}}{{\bar{\varepsilon }}_{D}^{p}(\eta ,{\dot{\bar{\varepsilon }}}^{p})}=1$$

where ${\bar{\varepsilon }}^{p}$ is the plastic strain and ${\bar{\varepsilon }}_{D}^{p}$ is the strain at the onset of damage. Considering ${\bar{\varepsilon }}_{D}^{p}$ as a function of stress triaxiality η and plastic strain rate ${\dot{\bar{\varepsilon }}}^{p}$,

(7) $$\eta =\frac{-p}{q}$$

with *p* corresponding to the pressure stress and *q* to the Mises equivalent stress. When the previous condition is met, the material enters the third stage, degradation, where it begins to lose mechanical properties until final failure.

(8) $${\bar{\varepsilon }}_{D}^{p}=\left[D_{1}+D_{2}\cdot e^{\left(-D3\cdot \eta \right)}\right]\cdot \left[1+D_{4}\cdot \ln\left(\frac{{\dot{\bar{\varepsilon }}}^{p}}{{\dot{\bar{\varepsilon }}}_{0}}\right)\right]\cdot \left[1+D_{5}\cdot \left(\frac{T-T_{0}}{T_{m}-T_{0}}\right)\right]$$

When damage initiates, the material stiffness degrades continuously, as described by Equation (8), until failure [51]. *D*_1_ suggests a finite strain, even at high stress triaxiality, taking as reference experimental data from Charpy tests (where stress triaxiality reaches high values, eliminating the influence of *D*_2_ and *D*_3_), at a reference strain rate (excluding the influence of *D*_4_), and at room temperature (annulling *D*_5_ and temperature dependence) [57]. *D*_2_ and *D*_3_ are retrieved from experimental analysis at a known strain rate and room temperature [58]. *D*_4_ and *D*_5_ are obtained from experimental data, as reported in the study of the influence of strain rate and temperature on PEEK through Taylor tests [59].

Although the printed parts are characterized at the macroscopic level primarily by robust anisotropy, the material modeling was performed at the microscale. For this reason, the material is considered isotropic at the numerical level, with the parts’ anisotropy introduced through filament orientation, as reflected in the geometries used.

Once a section of the material meets the failure point, the chip is torn out. This is numerically emulated by element deletion: when an element’s energy reaches the fracture energy, it is removed. This creates a discontinuity in a continuous medium and physically separates a solid into two parts, which is necessary in this application to simulate machining and chip removal.

Calculated from the stress–strain curve, the fracture energy can be identified as the area under the curve from the initial plastic strain ${\bar{\varepsilon }}_{0}^{p}$ to the final plastic strain ${\bar{\varepsilon }}_{f}^{p}$ multiplied by the characteristic length of the element *L_c_* [60].

(9) $$G_{f}=\int_{{\bar{\varepsilon }}_{0}^{p}}^{{\bar{\varepsilon }}_{f}^{p}}L_{c}\cdot \sigma_{y}\cdot d{\bar{\varepsilon }}^{p}=\int_{0}^{{\bar{u}}_{f}^{p}}\sigma_{y}\cdot d{\bar{u}}^{p}$$

The parameters and mechanical properties of the PEEK used in the analysis are compiled by Daniel et al. [58] and shown in Table 3.

Using Equation (9) and the parameters shown in Table 3, the fracture energy can be calculated numerically, yielding a value of 0.3086 N/mm (308.56 J/m^2^).

#### 3.2.3. Tool–Workpiece Interaction

The tool–workpiece contact was implemented using a surface-to-surface contact formulation with a Coulomb friction coefficient of *μ* = 0.25 between the tungsten carbide tool and the PEEK workpiece. Thermal dissipation in the numerical model was assumed to be entirely driven by plastic deformation, because the heat generated by plastic deformation is orders of magnitude higher than that derived from friction during chip formation via the inelastic heat fraction, setting the Taylor–Quinney coefficient to *β* = 0.9, while direct frictional heat generation at the tool–chip interface was omitted.

Furthermore, although material adhesion is a relevant physical phenomenon in PEEK machining, its explicit modeling was excluded due to its high numerical complexity and computational cost. Because this study focuses on the global cutting dynamics of PEEK rather than isolated adhesion events, this simplification was appropriate and was validated by the strong agreement between the numerical predictions and experimental results.

## 4. Results

This section presents the computational results, benchmarked against experimental data from previous research. Although the literature lacks specific studies on 3D-printed PEEK micromachining, orthogonal cutting models remain a well-established method for analyzing machining mechanics. Research into molded or carbon-fiber-reinforced polymers, though distinct from the current material, consistently identifies cutting speed, depth of cut, and printing orientation as critical parameters. These factors significantly influence machining forces and thermal gradients, which in turn dictate the risk of delamination and poor surface finish [61,62]. Therefore, this study first compares and develops orientations to validate the numerical model and provides a consistent analysis. Following this validation, the scope is expanded to simulate intermediate angles and evaluate the influence of printing sequences on performance.

### 4.1. Model Validation

**Figure 6:**
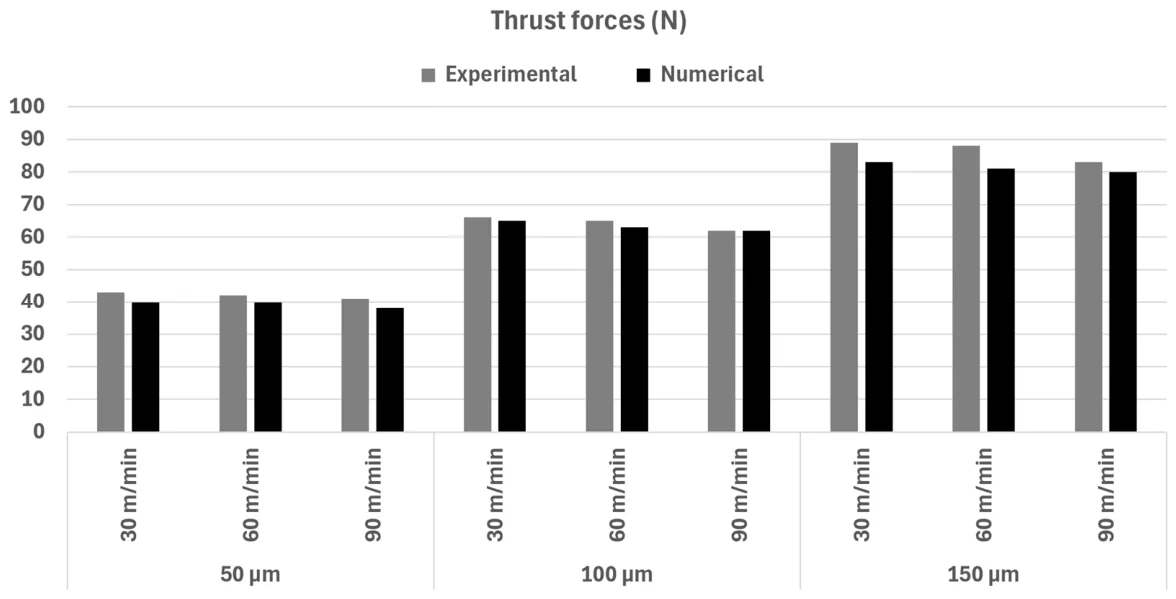
Experimental and numerical comparison of cutting forces for the case of 45°/−45°.

The validation of the computational framework was performed by comparing the predicted reaction forces against experimental data for the 0° and 45°/−45° printing orientations. As shown in Figure 6 for the 45°/−45° case, the numerical model shows high predictive accuracy, with errors below 9% across all tested conditions. While the numerical results consistently underestimate the experimental peaks slightly, the mean error remains low at approximately 9%. The results indicate that cutting forces are primarily driven by the depth of cut, with a significant increase from 50 μm to 150 μm due to the larger volume of material being removed. Conversely, a subtle inverse relationship is observed between cutting speed and force: as speed increases, force decreases slightly. This trend suggests some thermal softening in the PEEK material, though this effect is secondary to the influence of the depth of cut. At intermediate depths, such as 100 μm, this variation is marginal and occasionally obscured by experimental signal noise.

**Figure 7:**
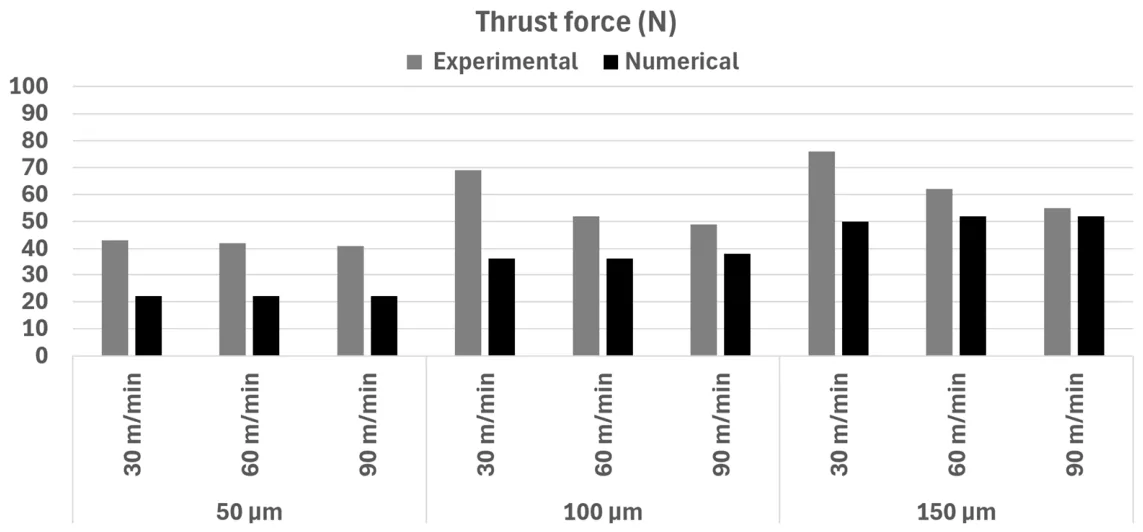
Comparison between experimental and numerical cutting-force signals for the 0 ° case.

In contrast, the results for the 0° building orientation (Figure 7) show a more pronounced discrepancy between numerical predictions and experimental measurements, with error values reaching approximately 50%. This divergence is partly due to the comparison methodology, which compares mean force values from numerical simulations with peak force values from experimental data. However, a closer analysis of the raw experimental signals suggests that this deviation is heavily influenced by material adhesion to the cutting tool, a phenomenon previously documented in [48]. When accounting for adhesion effects and comparing underlying signal attributes rather than just peak values, the correlation between the numerical model and experimental behavior becomes much clearer. The computational model does not account for material adhesion to the tool, so the tool shape remains invariant throughout the machining process.

**Figure 8:**
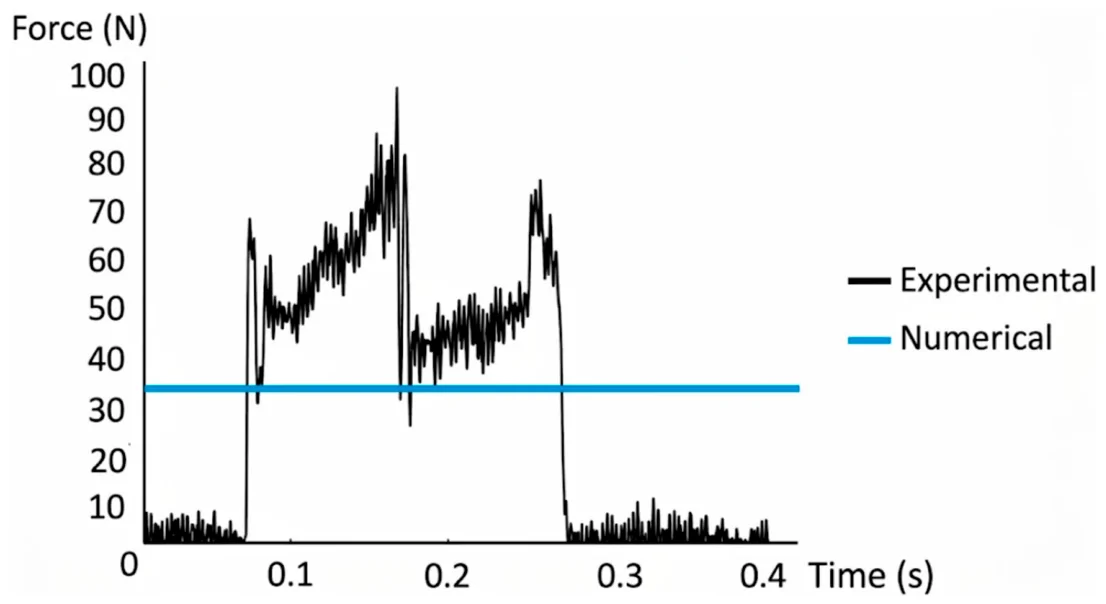
Representation of the raw experimental signal versus the average numerical cutting forces.

As documented in [48], the machining process in this orientation is characterized by a cyclic accumulation of material adhering to the tool tip. This build-up continues until the adhered material is abruptly torn away, causing a sharp, severe drop in force values, as shown by the experimental peaks and valleys in Figure 8. The material removal mechanism specific to this orientation, which will be analyzed in subsequent sections, further exacerbates this behavior. When the numerical data (Figure 8) is compared against the lower threshold of the raw experimental signals rather than the peaks, a much stronger correlation emerges. Adhesion complicates error analysis by introducing stroke-to-stroke instabilities and significant signal noise. This phenomenon must be considered, as it significantly alters machining forces by modifying the tool’s pristine geometry through material adhesion driven by high localized temperatures. Given the complexity of incorporating this phenomenon into the numerical model, the comparison between experiments and simulations was conducted using the lowest point of the experimental signal. This region corresponds to a state where chip adhesion does not affect the tool geometry; therefore, it directly correlates with the numerical results.

Notably, the highest discrepancies occur at lower machining intensities (e.g., 30 m/min and 50 μm), where adhesion effects dominate the signal. Conversely, the error is minimized at higher parameters (90 m/min and 150 μm), suggesting that as the depth of cut and cutting speed increase, their mechanical influence begins to outweigh the signal noise caused by adhesion. Under these conditions, the error margins stabilize to levels comparable to the 45°/−45° case, following the same predictable trends and significantly reducing the associated numerical error.

To analyze the disparity in the values obtained at shallow cutting depths, we directly compare the complete signal recorded during machining. As can be seen in Figure 8, once the comparison is performed using signal correlation rather than peak values, it becomes evident that the material adhesion process alters the recorded cutting forces and that, to correlate the experimental and numerical data, it is necessary to use the minimum values of the experimental signal for the analysis. To establish a criterion for selecting a representative signal value without the influence of material adhesion, the average cutting force was calculated over a 0.05-s interval in the lower portion of the signal to reduce noise-induced error.

**Figure 9:**
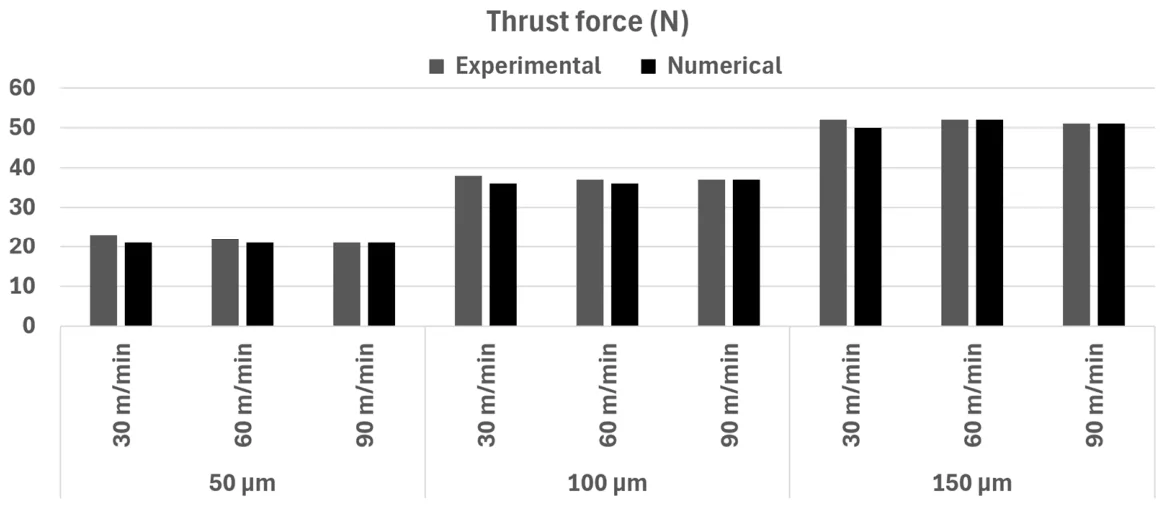
Comparison between experimental and numerical forces considering material adhesion influence for 0° building orientation samples.

A high degree of correlation (as shown in Figure 9) is achieved once the influence of material adhesion is accounted for by comparing numerical predictions with experimental values. Under this refined comparative framework, the numerical model yields results that closely match the experimental data, with a near-zero mean error across the full range of depths and speeds. Specifically, the model accurately captures the scaling of cutting forces from 50 μm to 150 μm, maintaining stability even as cutting speeds vary. This strong agreement confirms the model’s reliability in representing the underlying machining physics for the 0° orientation.

Consequently, the computational framework is considered validated, as the comparison of the numerical and experimental results from the 18 tests conducted shows an error of less than 9%, and will serve as the basis for the parametric studies and process variations explored in the following sections. As noted in the previous section, the numerical model does not account for the material’s anisotropic nature.

### 4.2. Further Analysis

After validating the model using the 0° and 45°/−45° cases presented in the previous section, the study examines the influence of printing orientation on machining forces. By analyzing individual orientations (0°, 90°, 45°, and −45°) alongside multi-layer sequences (0°/90°and 45°/−45°), the goal is to identify specific mechanical synergies and fracture behaviors that emerge during orthogonal machining of 3D-printed structures. Regarding the thermal analysis, the numerical model assumes plastic deformation of the workpiece as the primary heat source. These results, presented below, provide clear evidence of the process physics despite the limited scope of the heat-source modeling.

#### 4.2.1. Influence of Building Orientation

**Figure 10:**
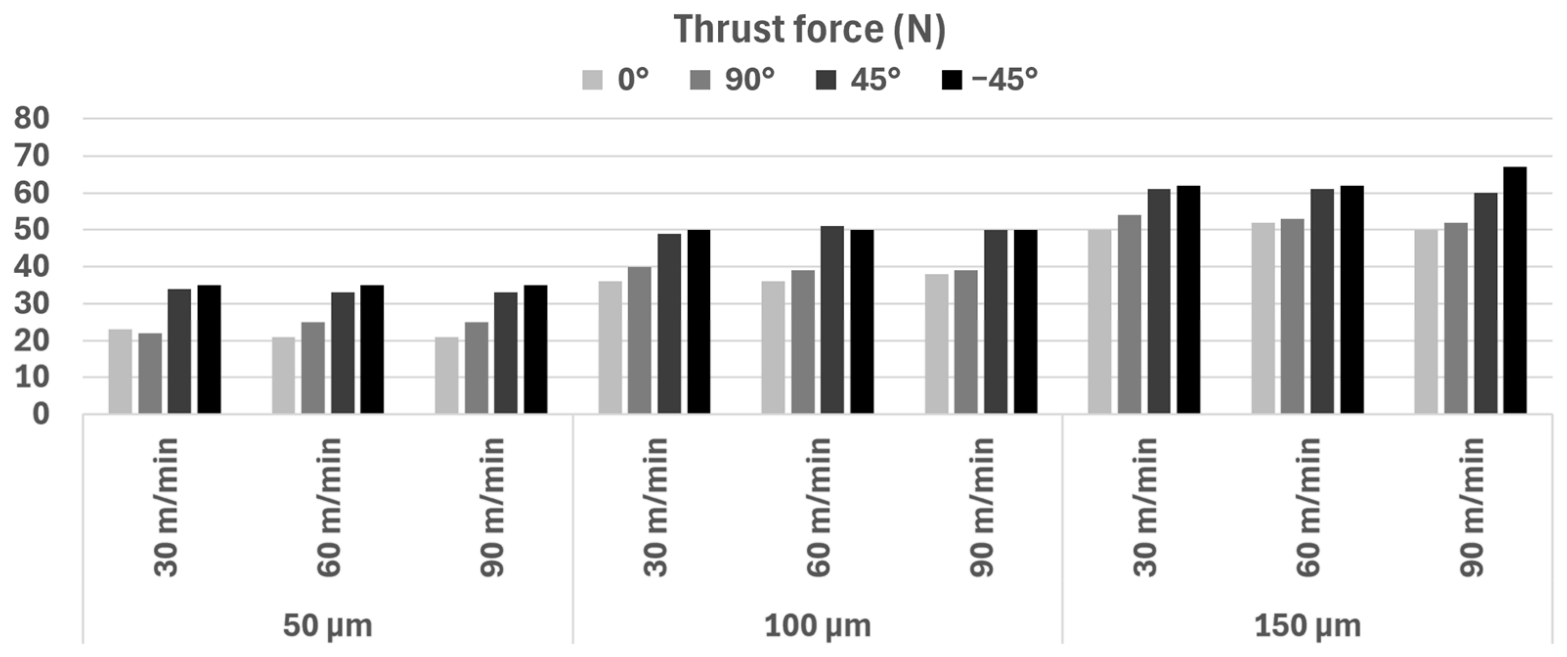
Cutting force values for the tests conducted at 0°, 90°, +45°, and −45°.

**Figure 11:**
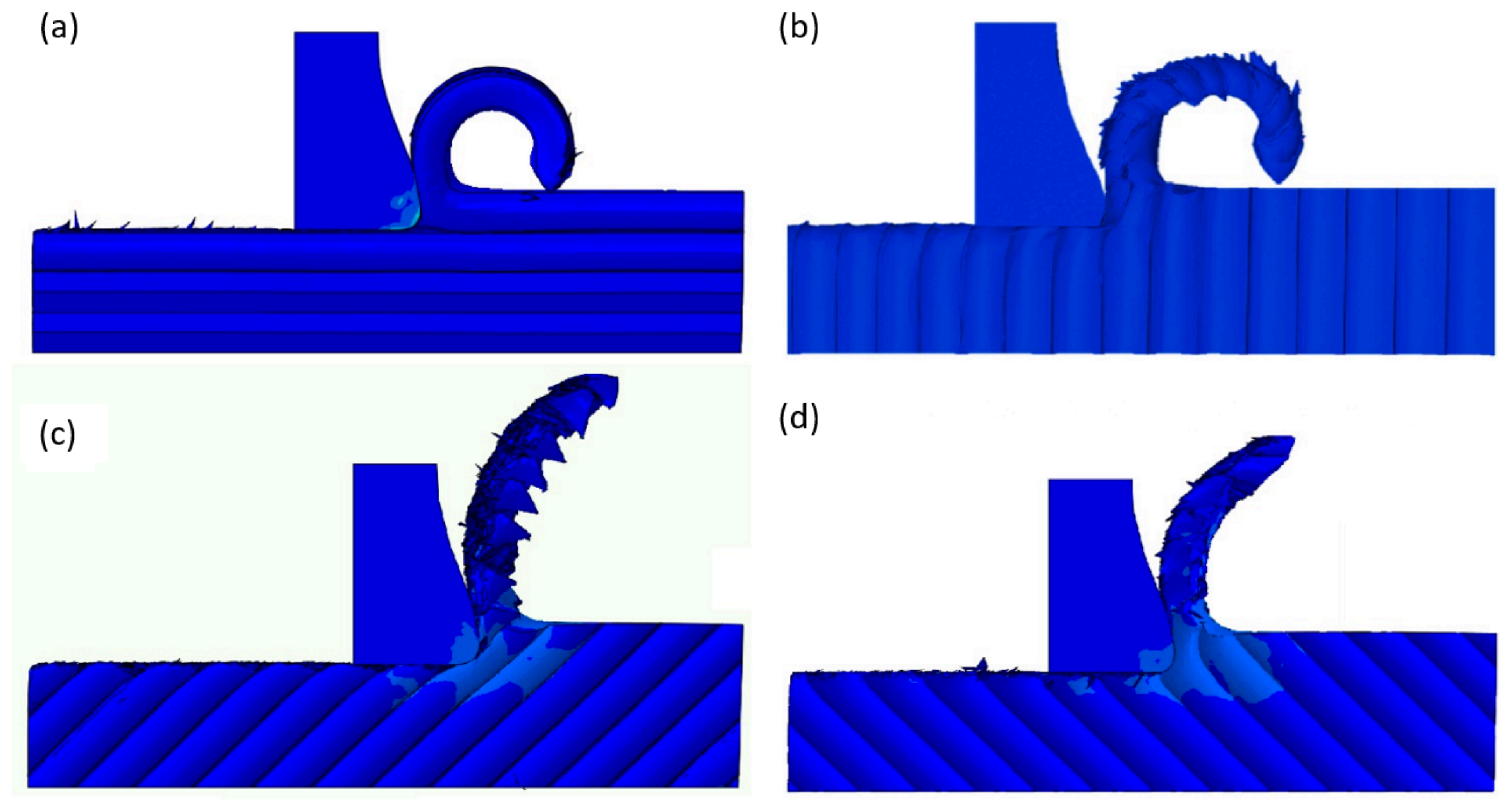
Machining samples at 30 m/min and 150 μm for different building orientations: (**a**) 0°. (**b**) 90°. (**c**) 45°. (**d**) −45°.

Figure 10 details the influence of building orientation on reaction forces, with the corresponding chip formation mechanisms illustrated in Figure 11. Across all cases, the depth of cut remains the primary driver of cutting force magnitude. However, cutting speed and raster orientation reveal the significant anisotropy inherent in FFF-printed PEEK.

The relationship between cutting speed and force varies across samples, as the specific building orientation dictates which physical mechanisms dominate the process. For the 0∘ orientation, cutting speed has a nearly negligible influence. Force decreases slightly as speed increases. This behavior is primarily due to localized thermal softening, in which heat generated at the tool tip reduces the material’s flow stress. In contrast, the 90°, 45°, and −45° orientations show a direct correlation: cutting forces increase with speed. In these configurations, the mechanical resistance, driven by specific chip-formation dynamics and strain-hardening effects, effectively outweighs any thermal softening, leading to higher energy requirements at greater velocities.

The printing orientation significantly dictates the energy required for material removal:

- 0° (Minimum Force): This configuration exhibits the lowest forces as material removal occurs primarily through filament delamination. Interfacial porosity acts as a stress concentrator, facilitating separation with minimal plastic deformation (Figure 11a).
- 90°: Forces increase slightly as the tool cuts transversely across the filaments, requiring higher localized stress to achieve fracture (Figure 11b).
- 45°/−45° (Maximum Force): These orientations require the highest energy input due to the larger volume of material undergoing deformation before chip formation (Figure 11c,d).
- −45° (Critical Case): This represents the most unfavorable scenario. The filament orientation directly opposes the shear plane, maximizing mechanical resistance and the work required for tool advancement.
- As shown in Figure 11, the specific layer deformation and the resulting chip morphologies reflect variations in mechanical resistance with inclination angles.

#### 4.2.2. Impact of Printing Stacking Sequence

To maximize mechanical performance, 3D-printed structures typically employ a combination of filament orientations rather than a unidirectional layer-by-layer approach. Consequently, the specific 3D printing sequence becomes a critical design variable that requires detailed analysis to optimize components for their intended applications. This section evaluates the influence of combining different building orientations, as the interaction between layers, each possessing distinct relationships between cutting speed, depth of cut, and force, induces unique mechanical synergies that alter the overall machining behavior. To complement the force analysis, this study also examines the thermal profile of the machined surface. Because temperature strongly dictates the machinability of thermoplastics, this study prioritizes the thermal gradients at the tool–workpiece interface. By correlating experimental and numerical temperature data with the observed cutting forces, the study establishes a more comprehensive understanding of the material’s response under varying laminate configurations.

- 45°/−45°

**Figure 12:**
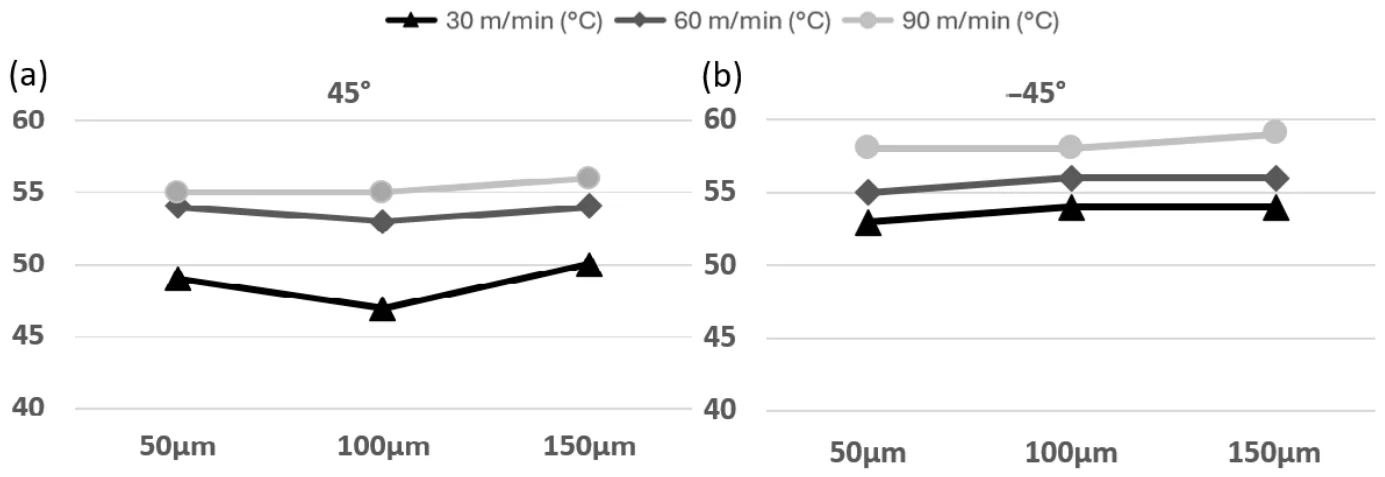
Temperature data subtracted from the numerical modeling for the building orientations of: (**a**) 45° and (**b**) −45°.

To establish a baseline for the stacking sequence analysis, the 45° and −45° orientations were examined first. Figure 12 shows that surface temperatures remain relatively insensitive to changes in the depth of cut. In contrast, cutting speed appears to show a clear influence; although the values remain within a narrow range, a consistent upward trend is observed as speed increases. This behavior is linked to the previously discussed stress states, in which the building orientation dictates the magnitude of the machining forces, thereby modulating the material’s sensitivity to other parameters. The rise in cutting forces is strongly coupled with depth of cut and temperature, creating a negative effect: increased depth of cut raises machining forces, thereby elevating temperatures. This thermal rise softens the PEEK material, promoting adhesion to the tool and further increasing the forces, ultimately degrading the surface finish. Notably, the −45° orientation, identified in previous sections as causing greater mechanical deformation, contributes to higher heat generation.

**Figure 13:**
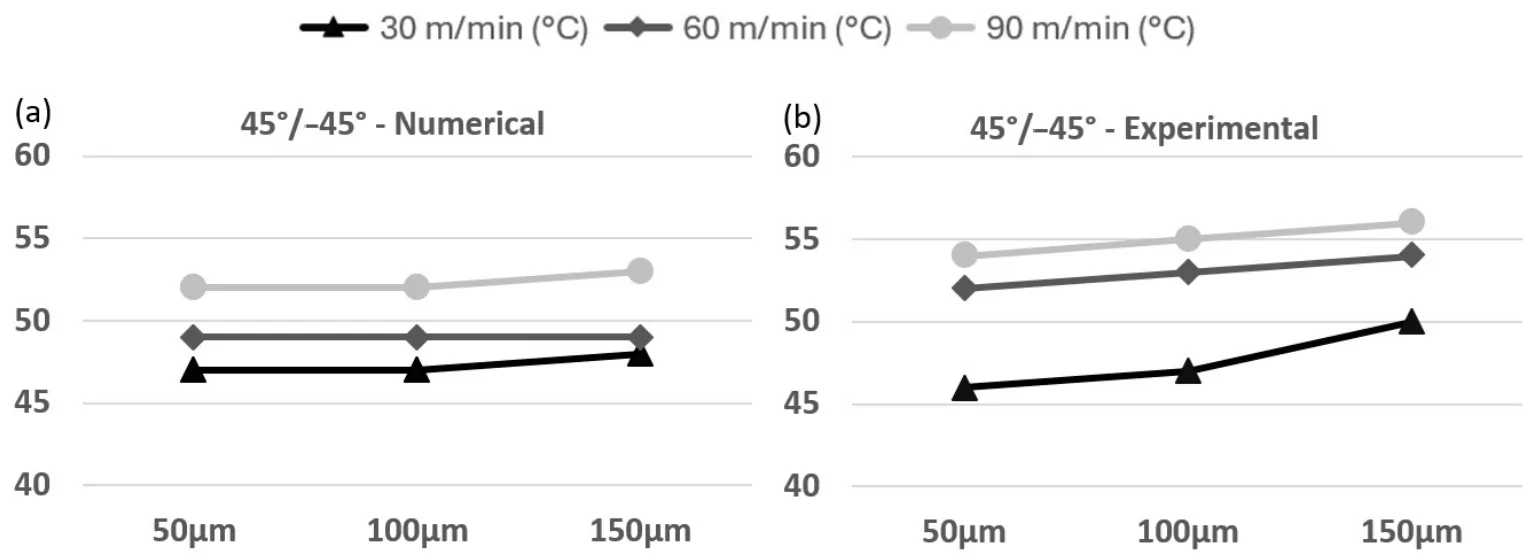
Temperature values for 45°/−45° building orientation for: (**a**) numerical analysis and (**b**) experimental tests.

The stacking-sequence influence was evaluated by comparing numerical and experimental data for the 45°/−45° laminate (Figure 13). Both methods yield consistent results regarding the impact of machining conditions. While these results are generally comparable to those of the standalone 45° and −45° specimens, the 45°/−45° layup shows a unique, albeit slight, upward trend in temperature with increasing depth of cut. Although the numerical model does not fully capture the magnitude of this depth-dependent influence, it accurately elucidates the impact of cutting speed, with lower temperatures recorded at lower velocities, in alignment with experimental observations.

**Figure 14:**
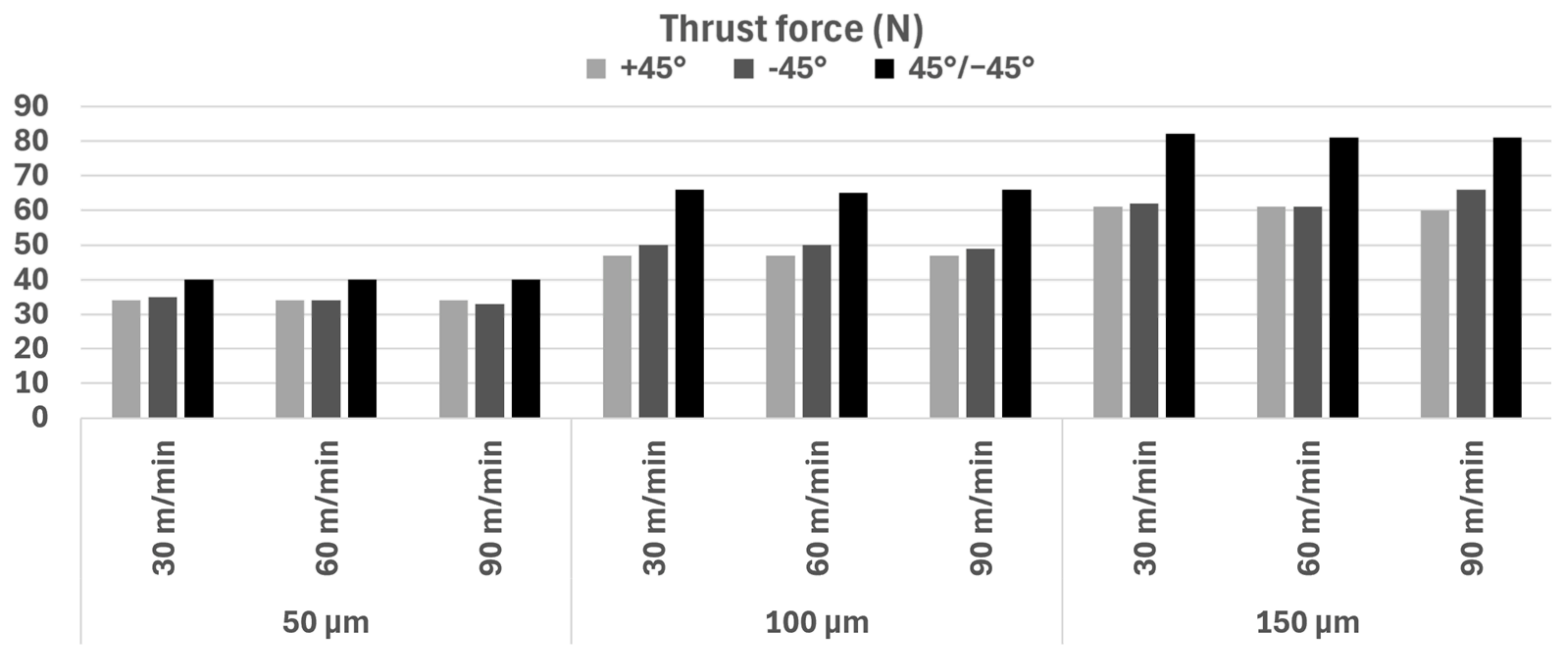
Average cutting force values obtained from the numerical analysis for 45°, −45°, and 45°/−45°.

Regarding mechanical loads, Figure 14 shows that the 45°/−45° laminate generates higher cutting forces than either orientation alone. This may be attributed to increased structural rigidity within the composite layup, which restricts material flow compared to monolithic samples.

**Figure 15:**
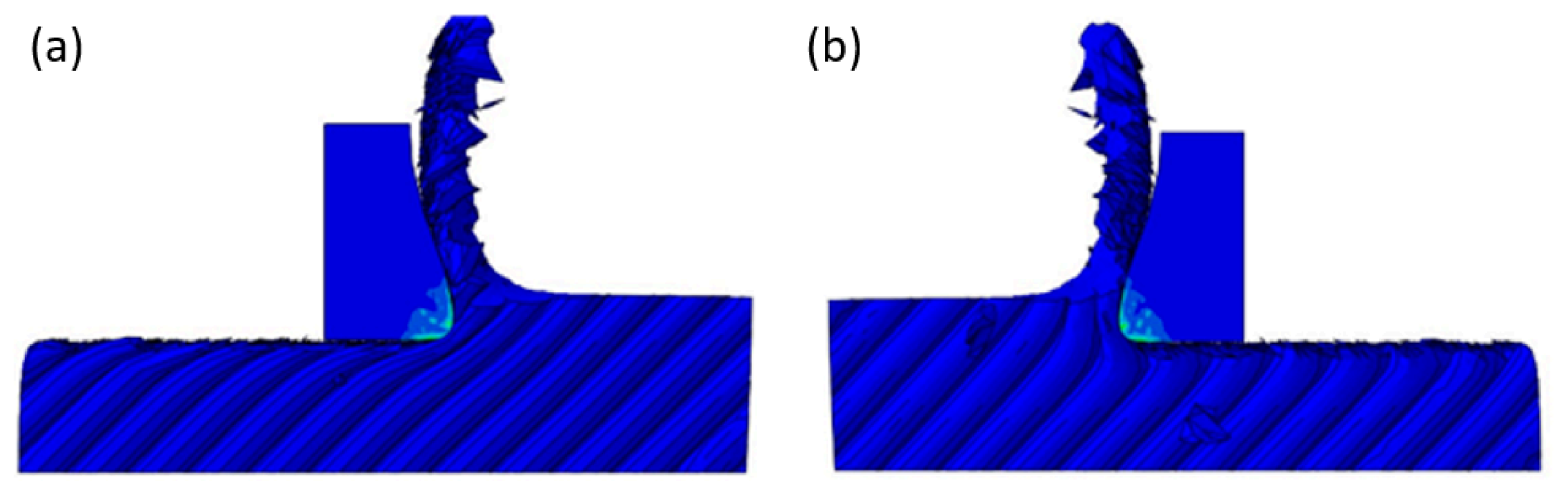
45°/−45° laminate machining process at 30 m/min and 150 μm with (**a**) 45° side view and (**b**) −45° side view.

Figure 15 further supports this phenomenon by showing the chip morphology. The chip formation in the 45°/−45° laminate differs significantly from the unidirectional patterns shown in Figure 11, confirming that the interlaced layers alter fracture mechanics. Furthermore, cutting parameters and force are positively correlated, where higher cutting speeds and greater depths of cut consistently result in higher reaction forces due to the enhanced mechanical resistance of the 3D-printed structure.

- 0°/90°

**Figure 16:**
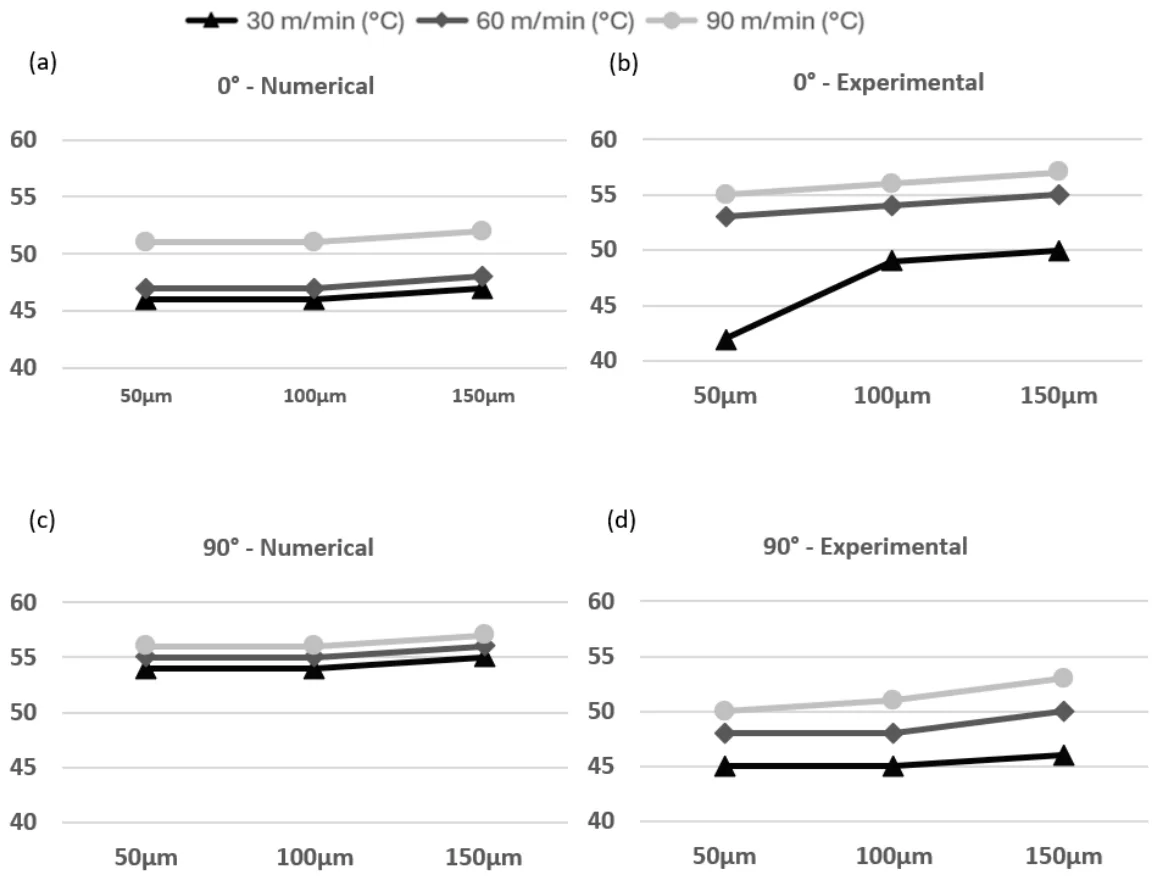
Temperature comparison for 0° building orientation between: (**a**) numerical analysis and (**b**) experimental tests. Numerical temperature data for the cases of: (**c**) 90° and (**d**) 0°/90°.

As with previous cases, the thermal response of the 0°/90° specimens is strongly influenced by cutting speed and depth of cut. Figure 16 illustrates that the comparison between numerical and experimental results for this printing configuration follows the trends observed in the 45°/−45° laminate, though the mechanical transitions are more extreme in this case. This is due to the alternating stress states between the pure shear experienced by the 90° filaments and the compression state of the 0° filaments.

As shown in Figure 16, this orientation combination also alters the absolute temperature range. Interestingly, the overall machining temperatures for the 0°/90° laminate are lower than those of the unidirectional specimens. This reduction may be attributed to the increased presence of interfacial voids and the specific stacking morphology, which likely alters heat dissipation or generation during layer-by-layer material removal; this suggests a potential reduction in the interlayer thermal barrier by introducing micro-voids in various directions, thereby potentially allowing better heat dissipation.

Figure 16 also shows the significant influence of machining speed on the aforementioned heat dissipation. As cutting speeds increase, the recorded temperature rises. This is because, as cutting speed increases, the time available for heat dissipation decreases, resulting in a local temperature increase in the machining area of up to 14%.

**Figure 17:**
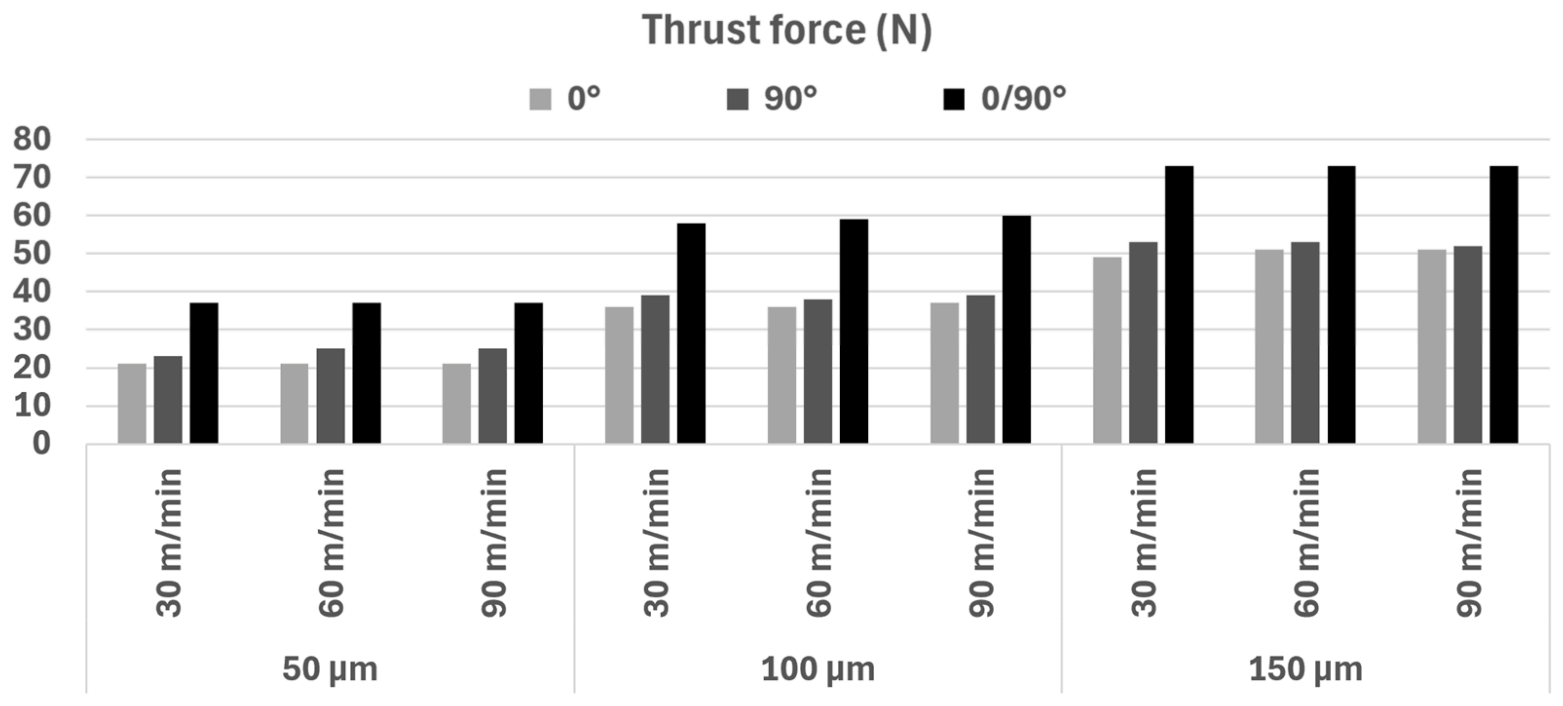
Cutting force values for the tests conducted for 0°, 90°, and 0/90°.

**Figure 18:**
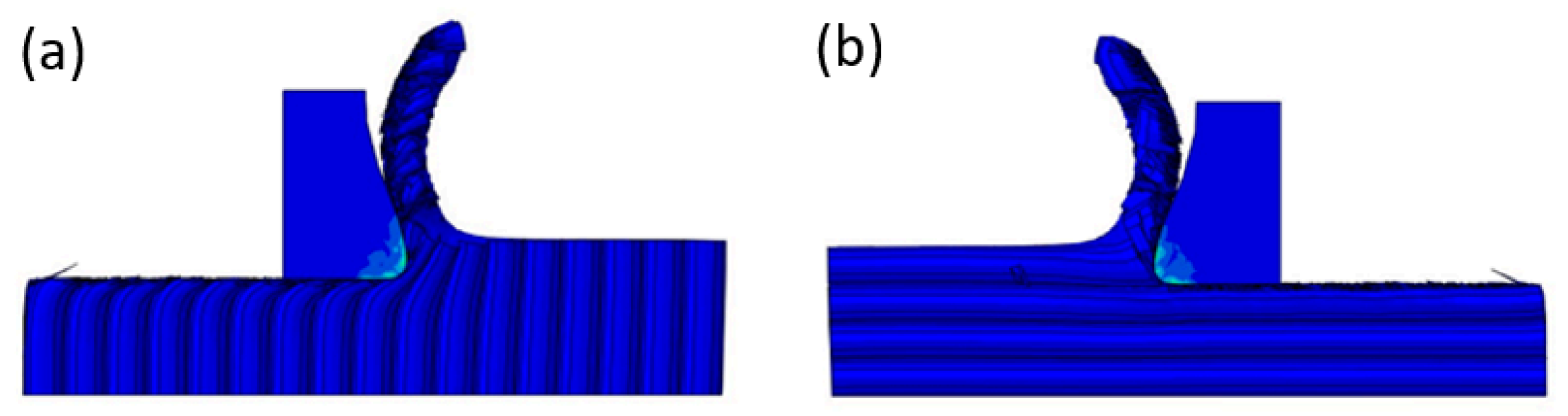
0°/90° laminate machining process at 30 m/min and 150 μm for (**a**) 90° and (**b**) 0° side views.

The mechanical load results for this layup indicate a significant increase in cutting forces compared to the individual 0° and 90° cases (Figure 17). This behavior suggests a positive relation regarding structural performance. The pure shear state that dominates the 90° orientation is partially counteracted by the compressive resistance of the 0° filaments. This reinforcement improves the overall mechanical properties of the 3D-printed structure, as shown by the stable chip formation in Figure 18. Furthermore, the lower temperatures used in this printing strategy contribute to these higher force requirements. By reducing thermal softening, which was more prevalent in the monolithic samples, the material retains higher flow stress, requiring greater force to perform the machining process.

## 5. Conclusions

This research investigated the complex interplay between additive manufacturing parameters and the subsequent machinability of 3D-printed PEEK through experimental validation and numerical modeling. The outcomes of the current study lead to the following primary conclusions.

- Impact of Anisotropy on Machining Forces: Building orientation significantly dictates the energy required for material removal. The 0° orientation requires the lowest forces due to filament delamination, while the −45° orientation is the most unfavorable scenario, maximizing mechanical resistance and shear-plane deformation.
- Dominance of Depth of Cut: Across all tested conditions and stacking sequences, the depth of cut remains the most influential machining parameter, directly governing chip formation and the magnitude of reaction forces.
- Thermal Softening: Increased cutting speeds correlate with higher localized temperatures, which can induce thermal softening, leading to a significant reduction in the mechanical properties of the material.
- Effect of material adhesion: This phenomenon is introduced at high temperatures and causes significant signal instability and degraded surface quality due to the alteration of the tool tip. This phenomenon introduces vibrations due to irregular machining, which significantly reduces machined surface quality.
- Synergistic Effects of Stacking Sequences: Multi-layer lamination strategies (e.g., 45°/−45° and 0°/90°) demonstrate improved performance compared to unidirectional prints. These sequences increase structural rigidity and produce more stable chip formation.
- Thermal Management via Lamination: Interestingly, the 0°/90° stacking morphology was found to lower overall machining temperatures compared to monolithic specimens, likely due to altered heat dissipation through interfacial voids. This suggests that strategic printing sequences can mitigate the risks of thermal degradation during post-processing.

The validated numerical framework established in this study serves as a predictive tool for optimizing the manufacturing and finishing of 3D-printed PEEK components for high-precision applications, highlighting the novelty of this work in investigating high-speed micro-machining ranges. At these critical cutting speeds, significant physical phenomena such as temperature-induced material adhesion occur. This research shows that while numerical models can bypass explicit adhesion simulation for computational efficiency, accounting for its physical presence is crucial to correctly correlating experimental signals with numerical predictions.

## Figures and Tables

**Table 1:** Parameter values used for PEEK during the printing process.

Parameter	Value	Unit
Printing temperature	430	°C
Bed temperature	240	°C
Chamber temperature	210	°C
Infill	100	%
Printing speed	30	mm/s
Layer height	0.15	mm
Layer width	0.4	mm

**Table 2:** Material parameter values used for the carbide tool during the numerical modeling [1].

Parameter	Value	Unit
Conductivity (κ)	2 × 10^1^	$\frac{N}{s\cdot K}$
Density (ρ)	1.19 × 10^−8^	$\frac{\mathrm{ton}}{{\mathrm{mm}}^{3}}$
Young Modulus (E)	5.34 × 10^5^	MPa
Poisson coeff. (ν)	2.2 × 10^−1^	-
Specific heat (Cp)	1.50 × 10^8^	$\frac{N\cdot \mathrm{mm}}{\mathrm{ton}\cdot K}$

**Table 3:** Parameter values used for PEEK during the numerical modeling.

PEEK	Value	Unit
Conductivity (κ)	9.5 × 10^−1^	$\frac{N}{s\cdot K}$
Density (ρ)	1.10 × 10^9^	$\frac{\mathrm{ton}}{{\mathrm{mm}}^{3}}$
Young’s modulus (E)	2 × 10^3^	MPa
Poisson coeff. (ν)	3.8 × 10^−1^	−
Specific heat (Cp)	2.18 × 10^9^	$\frac{N\cdot \mathrm{mm}}{\mathrm{ton}\cdot K}$
Johnson–Cook hardening parameters [2]		
A	8 × 10^1^	MPa
B	1 × 10^1^	MPa
n	1.2	−
m	7 × 10^−1^	−
Tm	6.14 × 10^2^	K
Reference strain rate ${\dot{\bar{\varepsilon }}}_{0}^{p}$	1 × 10^−3^	s^−1^
C	3.4 × 10^−2^	−
Johnson–Cook damage parameters [2]		
D1	5 × 10^−2^	−
D2	1.2	−
D3	−2.52 × 10^−1^	−
D4	−9 × 10^−3^	−
D5	1	−
Fracture energy parameters *G_f_*		
*L_c_*	2.66 × 10^−2^	mm
*σ_y_*	8 × 10^1^	MPa
${\bar{\varepsilon }}_{0}^{p}$	6 × 10^−2^	−
${\bar{\varepsilon }}_{f}^{p}$	3.5 × 10^−1^	−

## Data Availability

Data will be made available on request.
